# A new method to determine optimum temperature and activation energies for enzymatic reactions

**DOI:** 10.1007/s00449-016-1596-7

**Published:** 2016-04-11

**Authors:** M. Wojcik, J. Miłek

**Affiliations:** Department of Chemical and Biochemical Engineering, Faculty of Chemical Technology and Engineering, University of Science and Technology, Seminaryjna 3, 85-326 Bydgoszcz, Poland

**Keywords:** Optimum temperature, Biokinetics, Enzyme activity, Enzyme deactivation, Kinetic parameters, Inulinase

## Abstract

A new method for determination of the optimum temperature and activation energies based on an idea of the average rate of enzymatic reaction has been developed. A mathematical model describing the effect of temperature on a dimensionless activity for enzyme deactivation following the first-order kinetics has been derived. The necessary condition for existence of the function extreme of the optimal temperature has been applied in the model. The developed method has been verified using the experimental data for inulinase from *Kluyveromyces marxianus*.

## Introduction

Determination of optimum temperature is commonly applied to characterize enzymes. It is based on the graph which shows the change in an enzyme’s activity with increasing temperature. Both the rate of chemical reaction, as well as the rate of enzyme deactivation, increase with increasing temperature. At moderate temperatures deactivation rate is negligible and can be ignored, while the rate of enzymatic reaction increases with temperature, as for any chemical reaction.

At higher temperatures the effect of enzyme deactivation on its final activity increases. When the reaction occurs at an optimum temperature, equilibrium between these two processes is established. In many cases [1–4] the measurements of activity are made every 10 °C and accuracy of the optimum temperature determination is limited. Some authors [5–7] demonstrate a relationship between the activity and temperature on Arrhenius diagram, and the intersection point of straight lines determines optimum temperature. Such determined optimum temperature has, however, limited significance since it depends upon assay duration. With increased time of assay the optimum is shifted toward lower temperatures. However, it is possible to make use of the optimum temperature for qualitative comparison of stability of enzymes from various sources, if such measurements have been carried out under identical conditions (substrate type and concentration, pH, etc.), and within similar reaction times.

Application of optimal temperature control in a batch bioreactor enables significant reduction of the reaction time [8], or the amount of the spent enzyme [9], when compared to an isothermal process. The advantages resulting from implementation of the optimal temperature control are the greater the higher is the value of the quotient of activation energy for enzyme deactivation process and that of reaction activation. The activation energies indispensable for optimization computations are determined experimentally or calculated from the activity graph. Saqib et al. [7] made use of activity determinations of endoglucanase for the uprising part of the activity curve by applying Arrhenius diagram, while thermostability was studied by maintaining the enzyme at the predetermined temperatures. Iqbal et al. [5] employed both parts of the activity curve for glucoamylase in estimating energies of enzyme activation and deactivation, respectively. However, accounting for experimental data in the vicinity extreme of the enzyme activity leads to considerable errors in estimating activation energies.

In modeling thermal stability and activity of inulinase Santos et al. [10] utilized a concept based on accounting for effects of time and temperature on the enzymatic process. In this concept kinetic parameters are related to the reaction rate at a fixed moment of the measurement, though due to enzyme deactivation gradual decrease of the reaction rate takes place, so in fact an average value of this rate is determined.

The method of Santos et al. [10] requires additional estimation of the starting parameters based on the measurements of the deactivation rate. The aim of the present work was to develop a mathematical model that would take into account the effects of time and temperature on enzyme activity determined using the concept of the average reaction rate. The model will be verified by means of the experimental data for inulinase [10, 15].

## Theory

During determination of the enzyme activity the experimental conditions are selected in such a way, that the effects of the substrate and product concentrations variations can be neglected. Thus the reaction rate *ν* can be described by Eq. (1)

1 $$\nu = - \frac{{{\text{d}}S}}{{{\text{d}}t}} = \frac{{{\text{d}}P}}{{{\text{d}}t}} = kE$$

where *S* is concentration of substrate, *P* is concentration of product, *t* is time, *k* is kinetic constant and *E* is active enzyme concentration.

The constant *k* varies with temperature and can be expressed by the Arrhenius equation

2 $$k = k_{0} \exp \left( { - E_{\text{a}} /RT} \right)$$

where *k*_0_ is pre-exponential factor, *E*_a_ is activation energy, *R* is gas constant and *T* is absolute temperature.

With the increasing temperature the enzyme deactivates and this effect should be taken into account. In many cases [11–14] the rate of enzyme deactivation is approximated with the first-order kinetics leading to the following differential equation:

3 $$\frac{{{\text{d}}E}}{{{\text{d}}t}} = - k_{\text{d}} E$$

with an initial condition *E*(*t* = 0) = *E*_0_, where *k*_d_ is deactivation constant and *E*_0_ is initial active enzyme concentration.

Solution of Eq. (3) yields

4 $$E = E_{0} \exp \left( { - k_{\text{d}} t} \right)$$

A dependence between deactivation constant* k*_d_ and temperature is also given by the Arrhenius equation

5 $$k_{\text{d}} = k_{\text{d0}} \exp \left( { - E_{\text{d}} /RT} \right)$$

where *k*_d0_ is pre-exponential factor, *E*_d_ is activation energy for enzyme deactivation.

Final expression for an instantaneous reaction rate is obtained after substituting Eqs. (2), (4) and (5) in Eq. (1)

6 $$\nu = - \frac{{{\text{d}}S}}{{{\text{d}}t}} = \frac{{{\text{d}}P}}{{{\text{d}}t}} = k_{0} E_{0} \exp \left( { - \frac{{E_{\text{a}} }}{RT}} \right)\exp \left[ { - \left( {k_{{{\text{d}}0}} \exp \left( { - \frac{{E_{\text{d}} }}{RT}} \right)t} \right)} \right]$$

Depending on the applied analytical method enzyme activity is determined from the measurements of the rate of the substrate concentration decrease or by the rate of the product concentration increase. For the assay time *t*_*a*_ the average reaction rate $\bar{\nu }(T)$ is calculated after integrating Eq. (6)

7 $$\bar{\nu }(T) = \frac{{k_{{_{0} }} E_{{_{0} }} }}{{k_{{{\text{d}}0}} t_{\text{a}} }}\exp \left( {\frac{{E_{\text{d}} - E_{\text{a}} }}{RT}} \right)\left\{ {1 - \exp \left[ { - k_{\text{d0}} t_{\text{a}} \exp \left( { - \frac{{E_{\text{d}} }}{RT}} \right)} \right]} \right\}$$

A temperature at which a maximum value of the average reaction rate has been obtained is assumed as optimal. Usually a dimensionless enzyme activity, *a*, being a quotient of the reaction rate at a given temperature and that at an optimum temperature, is applied

8 $$a = \frac{{\bar{\nu }(T)}}{{\bar{\nu }(T_{\text{opt}} )}}$$

A dependence of dimensionless activity on temperature *T* can be expressed by the following equation

9 $$a = \frac{{\exp \left( {\frac{{\left( {E_{\text{d}} - E_{\text{a}} } \right)\left( {T_{\text{opt}} - T} \right)}}{{R \ T \ T_{\text{opt}} }}} \right) \ \left\{ {1 - \exp \left[ { - k_{{{\text{d}}0}} \ t_{\text{a}} \ \exp \left( { - \frac{{E_{\text{d}} }}{RT}} \right)} \right]} \right\}}}{{1 - \exp \left( { - k_{{{\text{d}}0}} \ t_{\text{a}} \ \exp \left( { - \frac{{E_{\text{d}} }}{{RT_{\text{opt}} }}} \right)} \right)}}$$

In order for a maximum activity to be achieved the necessary condition should be fulfilled, i.e.

10 $$\left. {\frac{{{\text{d}}a}}{{{\text{d}}T}}} \right|_{{T = T_{\text{opt}} }} = 0$$

After differentiation and substitution *T* = *T*_opt_ we obtain for constant denominator

11 $$\begin{aligned} \left( { - \frac{1}{{T_{\text{opt}}^{2} }}} \right) \ \frac{{\left( {E_{\text{d}} - E_{\text{a}} } \right)}}{R} \, \ \left( {1 - \exp \left( { - k_{{{\text{d}}0}} \ t_{\text{a}} \ \exp \left( { - \frac{{E_{\text{d}} }}{{RT_{\text{opt}} }}} \right)} \right)} \right) \hfill \\ + \frac{{E_{\text{d}} }}{{RT_{\text{opt}}^{2} }} \ k_{{{\text{d}}0}} \ t_{\text{a}} \ \exp \left( { - \frac{{E_{\text{d}} }}{{RT_{\text{opt}} }}} \right) \ \exp \left[ { - k_{\text{d0}} \ t_{\text{a}} \ \exp \left( { - \frac{{E_{\text{d}} }}{RT}_{\text{opt}} } \right)} \right] = 0 \hfill \\ \end{aligned}$$

Multiplication by $RT_{\text{opt}}^{2}$ and division by $\exp \left[ { - k_{\text{d0}} \ t_{\text{a}} \ \exp \left( { - \frac{{E_{\text{d}} }}{RT}} \right)} \right]$ gives

12 $$\left( {E_{\text{d}} - E_{\text{a}} } \right) \ \left\{ {\exp \left[ {k_{{{\text{d}}0}} \ t_{\text{a}} \ \exp \left( { - \frac{{E_{\text{d}} }}{RT}} \right)} \right] - 1} \right\} = E_{\text{d}} \ k_{{{\text{d}}0}} \ t_{\text{a}} \ \exp \left( { - \frac{{E_{\text{d}} }}{RT}} \right)$$

For further considerations it is more convenient to derive a parameter *β*

13 $$\beta = k_{\text{d0}} t_{\text{a}} \exp \left( { - \frac{{E_{\text{d}} }}{{RT_{\text{opt}} }}} \right)$$

Combining Eqs. (12) and (13) gives

14 $$E_{\text{d}} - E_{\text{a}} = \frac{{E_{\text{d}} \ \beta }}{\exp \beta - 1}$$

After substituting Eqs. (13) and (14) into Eq. (9) we obtain

15 $$a = \frac{{\exp \left( {\frac{{\left( {T_{\text{opt}} - T} \right)}}{{RTT_{\text{opt}} }} \ \frac{{E_{\text{d}} \beta }}{{\left( {\exp \beta - 1} \right)}}} \right)\left\{ {1 - \exp \left[ { - \beta \exp \left( {\frac{{E_{d} \left( {T - T_{\text{opt}} } \right)}}{{RTT_{\text{opt}} }}} \right)} \right]} \right\}}}{{1 - \exp \left( { - \beta } \right)}}$$

Having an experimental relationship between dimensionless enzyme activity and temperature it is possible to find *T*_opt_, *β* and *E*_d_ directly from Eq. (15) using a non-linear regression. Rearranging Eq. (12) we get Eq. (16) which can be employed in determination of *E*_a_

16 $$E_{\text{a}} = E_{\text{d}} - \frac{{E_{d} \ \beta }}{\exp \, \beta - 1}$$

## Results and discussion

The elaborated method has been verified using the data for inulinase from *Kluyveromyces marxianus* [10, 15], which deactivates according to the first-order kinetics [10, 13, 14]. The highest activity was observed for inulinase from *K. marxianus* at 63 °C and pH 4.8 in 0.1 M sodium acetate buffer [10] and at 60 °C and pH 4.0 in 0.05 M citrate–phosphate buffer [15]. Constants in Eq. (15) were determined from non-linear regression by means of SigmaPlot 12.3 software.

**Fig. 1:**
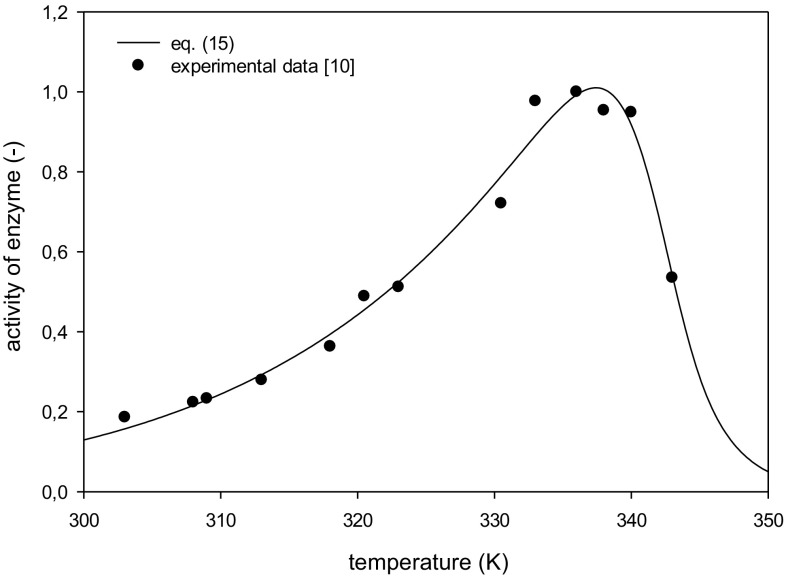
Activity variation of inulinase from *K. marxianus* for estimated kinetic parameters: *T*_opt_ = 337.27 ± 0.26 K, *β* = 0.2582 ± 0.0601, *E*_d_ = 393.40 ± 64.02 kJ mol^−1^

**Fig. 2:**
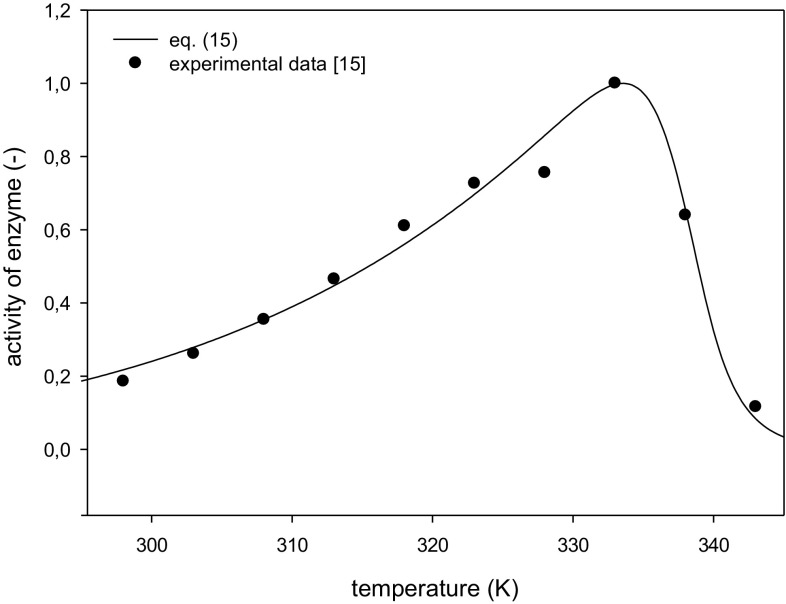
Activity variation of inulinase from *K. marxianus* for estimated kinetic parameters: *T*_*opt*_ = 333.55 ± 0.27 K, *β* = 0.1597 ± 0.0098, *E*_d_ = 479.74 ± 6.15 kJ mol^−1^

Very high values of the regression coefficient R^2^ were obtained 0.984 and 0.977 for data of Santos et al. [10] and Cazetta et al. [15], respectively. The experimental data points of inulinase activity and a dependence of its activity on temperature calculated based on the estimated parameters are plotted in Figs. 1 and 2. Very good agreement between the experimental data and Eq. (15) can be seen.

The calculated values of the constants in the mathematical model [Eqs. (15), (16)] and those determined by Santos et al. [10] and Cazetta et al. [15] are given in Table 1.

**Table 1:** Values of parameters estimated from mathematical model [Eqs. (15), (16)] and those presented in literature

Figure 1	Santos et al. [10]	Equations (15) and (16)
*T*_opt_ (K)	336	337.4
*E*_a_ (kJ mol^−1^)	45.1	48.6
*E*_d_ (kJ mol^−1^)	509.3	393.4

Figure 2	Cazetta et al. [15]	Equations (15) and (16)
*T*_opt_ (K)	333	333.6
*E*_a_ (kJ mol^−1^)	–	37.2
*E*_d_ (kJ mol^−1^)	–	479.7

### Optimum temperature

Optimum temperature given by Santos et al. [10] at 336 K has been assumed based on a few experimental measurements of enzyme activity at the temperatures in the vicinity of a maximum activity of inulinase, thus the error of the optimum temperature determination did not exceed 1.5 K. The value of the optimum temperature estimated from the model that takes into account the necessary condition for existence of function extreme is within this temperature range. The optimum temperature for inulinase from *K. marxianus* has been determined several times, and its value is between 328 K [16] and 343 K [17] when the activity measurements are performed during hydrolysis of 2 % sucrose aqueous solution. The estimated value of optimal temperature for experimental data of Cazetta et al. [15] was found to be 333.6 K which is well within the range of values reported in the literature.

Due to lack of data on the duration time of the activity measurements, it is not possible to assess whether the observed differences are caused only by the various thermostabilities of the applied enzyme preparations. Presented computations univocally revealed that all published information concerning the effect of temperature on the enzymes activity should contain data on the duration time of activity determination. Henceforth more detailed assessment of enzyme thermostability would be possible.

### Activation energy *E*_a_

Activation energy of the reaction estimated from Eq. (16) is about 10 % higher than that determined by Santos et al. [10]. This results from the fact that in the model lower increase in enzyme activation, while approaching to a maximum temperature and resulting in enhanced deactivation has been accounted for. A value of the activation energy for inulinase equals 49.7 kJ mol^−1^ and is within the values of 45.1 and 56.2 kJ mol^−1^ reported by Singh et al. [18] and Treichel et al. [14], respectively. Activation energy for inulinase prepareation used by Cazetta et al. [15] was found to be 37.2 kJ mol^−1^ which was a little lower than reported in the literature.

### Activation energy for enzyme deactivation E_d_

The values of the activation energy for enzyme deactivation estimated from the model were 393.4 and 479.7 kJ mol^−1^ for experimental data of Santos et al. [10] and Cazetta et al. [15], respectively. Mazutti et al. [19] studied directly deactivation of inulinase and have found value 343.9 kJ mol^−1^. Very high value of the activation energy (509.3 kJ mol^−1^) determined by Santos et al. [10] is most likely overestimated since the time of enzyme half-life calculated and determined experimentally are in agreement only at 50 °C. For the temperatures ranging from 52.5 to 60 °C deviations amount from 87 to 429 % (Table 2 in Santos et al. [10]).

## Conclusions

A new method for determination of the optimum temperature and activation energy based on a mathematical model that takes into account effect of temperature on the rate of enzyme activation and deactivation has been presented. The model has been verified using the experimental data for inulinase from *K. marxianus*. Very high value of the correlation coefficient has been obtained while the parameters estimated by means of a non-linear regression are consistent with the earlier studies.

## List of symbols

*a*: Dimensionless enzyme activity
*E*: Active enzyme concentration (M)
*E*_0_: Initial active enzyme concentration (M)
*E*_a_: Activation energy (J mol^−1^)
*E*_d_: Activation energy for enzyme deactivation (J mol^−1^)
*k*: Kinetic constant (h^−1^)
*k*_0_: Pre-exponential factor (h^−1^)
*k*_d_: Deactivation constant (h^−1^)
*k*_d0_: Pre-exponential factor (h^−1^)
*P*: Concentration of product (M)
*R*: Gas constant (J mol^−1^ K^−1^)
*S*: Concentration of substrate (M)
*t*: Time (h)
*t*_a_: Time of assay (h)
*T*: Temperature (K)
*T*_opt_: Optimum temperature (K)
*β*: Parameter described Eq. (13)
*ν*: Reaction rate (M h^−1^)
$\bar{\nu }(T)$: Average reaction rate (M h^−1^)
